# Supplementary material for: Towards the restoration of ancient hominid craniofacial anatomy: Chimpanzee morphology reveals covariation between craniometrics and facial soft tissue thickness
Source: PLoS One. 2021 Jun 4;16(6):e0245760. doi: 10.1371/journal.pone.0245760 (PMC8177512; doi:10.1371/journal.pone.0245760)
Supplement: S1 Table — (DOCX) [file pone.0245760.s001.docx]

**S1 Table. List of specimens used in this study.**

| Cat No. | ID | Species | Specimen condition | Sex | Age | Source |
| --- | --- | --- | --- | --- | --- | --- |
| 366 | PRI-Cleo | *Pan troglodytes* | living | F | 9 | KUPRI |
| 367 | PRI-Pal | *Pan troglodytes* | living | F | 10 | KUPRI |
| 365 | PRI-Popo | *Pan troglodytes* | living | F | 27 | KUPRI |
| 364 | PRI-Pen | *Pan troglodytes* | living | F | 33 | KUPRI |
| 274 | PSI-Akira | *Pan troglodytes* | living | M | 34 | KUPRI |
| 467 | PRI-Mari | *Pan troglodytes* | living | F | 35 | KUPRI |
| 456 | PRI-Reiko | *Pan troglodytes* | living | F | 44 | KUPRI |
| 690 | TZ-Apple | *Pan troglodytes* | fresh | F | 30 | KUPRI |
| 659 | PRI-9803 | *Pan troglodytes* | fresh | M | 34 | KUPRI |
| 1523 | HCZ-Chieko | *Pan troglodytes* | fresh | F | 40 | KUPRI |
| 1486 | PRI-10814 | *Pan troglodytes* | fresh | F | 43 | KUPRI |
| 573 | PRI-9783 | *Pan troglodytes* | fresh | F | 44 | KUPRI |
| 26 | KCZ-Yoko | *Pan troglodytes* | frozen | F | 20 | KUPRI |
| 318 | PRI-9262 | *Pan troglodytes* | frozen | M | 22 | KUPRI |
| 1320 | PRI-10301 | *Pan troglodytes* | frozen | M | 24 | KUPRI |
| 455 | PRI-9457 | *Pan troglodytes* | frozen | M | 32 | KUPRI |
| 788 | HAZ-Yuko | *Pan troglodytes* | frozen | F | 42 | KUPRI |
| 344 | PRI-9266 | *Pan troglodytes* | immersed | M | 29 | KUPRI |
| 485 | PRI-9473 | *Pan troglodytes* | immersed | M | 35 | KUPRI |
| M20189 | 38739.0 | *Pan paniscus* | wet specimen | M | 4 | Morphosource |
| – | – | *Homo sapiens* | living | M | 29 | Donated |
